# P2Y_2_ receptor decreases blood pressure by inhibiting ENaC

**DOI:** 10.1172/jci.insight.167704

**Published:** 2023-07-24

**Authors:** Antonio G. Soares, Jorge Contreras, Elena Mironova, Crystal R. Archer, James D. Stockand, Tarek Mohamed Abd El-Aziz

**Affiliations:** 1 Department of Cellular and Integrative Physiology, University of Texas Health Science Center at San Antonio, San Antonio, Texas, USA.; 2 Department of Integrative Biology and Pharmacology, University of Texas Health Science Center at Houston, Houston, Texas, USA.; 3 Zoology Department, Faculty of Science, Minia University, El-Minia, Egypt.

**Keywords:** Cell Biology, Nephrology, Hypertension, Ion channels, Sodium channels

## Abstract

Stimulating the Gq-coupled P2Y_2_ receptor (*P2ry2*) lowers blood pressure. Global knockout of *P2ry2* increases blood pressure. Vascular and renal mechanisms are believed to participate in *P2ry2* effects on blood pressure. To isolate the role of the kidneys in *P2ry2* effects on blood pressure and to reveal the molecular and cellular mechanisms of this action, we test here the necessity of the *P2ry2* and the sufficiency of Gq-dependent signaling in renal principal cells to the regulation of the epithelial Na^+^ channel (ENaC), sodium excretion, and blood pressure. Activating *P2ry2* in littermate controls but not principal cell–specific *P2ry2*-knockout mice decreased the activity of ENaC in renal tubules. Moreover, deletion of *P2ry2* in principal cells abolished increases in sodium excretion in response to stimulation of *P2ry2* and compromised the normal ability to excrete a sodium load. Consequently, principal cell–specific knockout of *P2ry2* prevented decreases in blood pressure in response to *P2ry2* stimulation in the deoxycorticosterone acetate–salt (DOCA-salt) model of hypertension. In wild-type littermate controls, such stimulation decreased blood pressure in this model of hypertension by promoting a natriuresis. Pharmacogenetic activation of Gq exclusively in principal cells using targeted expression of Gq–designer receptors exclusively activated by designer drugs and clozapine *N*-oxide decreased the activity of ENaC in renal tubules, promoting a natriuresis that lowered elevated blood pressure in the DOCA-salt model of hypertension. These findings demonstrate that the kidneys play a major role in decreasing blood pressure in response to *P2ry2* activation and that inhibition of ENaC activity in response to *P2ry2*-mediated Gq signaling lowered blood pressure by increasing renal sodium excretion.

## Graphical abstract

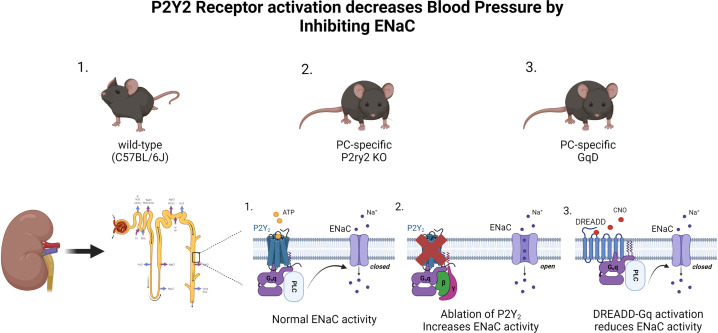

## Introduction

Cardiovascular disease is the leading cause of death in the United States and worldwide, responsible for about 20 million deaths per year (1). Hypertension is a major risk factor for cardiovascular disease, with risk increasing with higher blood pressure (2). In industrialized countries, approximately 90% of people will develop hypertension in their lifetimes (3). Currently, 1 of every 3 adults in America has high blood pressure, with the total number of Americans with hypertension well exceeding 75 million. High blood pressure is a symptom common to many distinct pathologies. This is so because control of blood pressure is complex and multifactorial, with diverse effects on different cells and tissues summating to influence blood pressure. Approximately 95% of the cases of hypertension are considered “essential hypertension”: hypertension of unknown etiology (4, 5). Due to the limited understanding of the causes of essential hypertension, often requiring continuous treatment involving costly combinatorial drug therapy, which in many instances is only of limited utility, for such therapy targets the symptom of elevated blood pressure rather than the causes of this elevation in blood pressure (6), the treatment and the consequences of this epidemic of hypertension account for the bulk of contemporary health care costs in America.

In parallel with neurogenic and endocrine regulation, paracrine pathways, to include purinergic signaling, contribute to the control of blood pressure (7–9). Purinoceptors are divided into 2 major classes. The nucleoside, adenosine, is the primary ligand for P1 receptors. Nucleotides, such as ATP and UTP, are the primary ligands for P2 receptors. P2 receptors are further subdivided into 2 distinct classes. P2X receptors are ionotropic ligand-gated ion channels. P2Y receptors are metabotropic GPCRs. Mammals express several P2Y receptor isoforms, including the P2Y_2_ receptor (*P2ry2*).

Activating *P2ry2* increases sodium excretion and decreases blood pressure (10–13). Knockout of *P2ry2* decreases sodium excretion and elevates blood pressure (10, 13, 14). Regulation of renal tubular transport has been proposed to be key to *P2ry2* effects on blood pressure (9–11). A direct link between tubular transport, systemic sodium balance, and blood pressure has long been established (15–17). Endocrine and paracrine regulation of discretionary sodium excretion happens in the distal renal nephron, to include the distal convoluted tubule, connecting tubule, and collecting duct. This discretionary sodium excretion fine-tunes plasma Na^+^ levels and plasma volume, and thus, blood pressure.

The epithelial Na^+^ channel (ENaC) is an ion channel in the degenerin/ENaC channel family that is highly selective for Na^+^ over other cations (18, 19). ENaC is expressed in the luminal plasma membranes of epithelial cells responsible for salt and water transport, including those in the kidneys (15, 20, 21). In the kidneys, ENaC is expressed in renal principal cells, which are localized to the late distal convoluted tubule, connecting tubule, and collecting duct system. The activity of ENaC is limiting for sodium reabsorption across the distal nephron. As such, ENaC is the final arbiter fine-tuning renal sodium excretion. In this capacity, ENaC functions as a key final effector of the renin-angiotensin-aldosterone system during the regulation of blood pressure. Moreover, because sodium reabsorption across principal cells is positively tied to K^+^ secretion from these cells, ENaC activity also influences serum potassium levels. Consequently, gain-of-function mutations in ENaC cause Liddle’s syndrome, autosomal dominant familial hypertension associated with hypokalemia and low renin and aldosterone levels (20, 21). The activity of ENaC in principal cells is under the discretionary control of peptide hormones and mineralocorticoid steroid hormones, including arginine vasopressin and aldosterone, respectively, and paracrine factors to include ATP.

ATP decreases ENaC activity by stimulating luminal P2Y_2_ receptors in principal cells (12, 13, 22–26). This activation of P2Y_2_ receptors stimulates phospholipase C (PLC) via Gq signaling, promoting the hydrolysis of membrane phosphatidylinositol 4,5-bisphosphate [PI(4,5)P_2_]. ENaC activity is dependent on membrane PI(4,5)P_2_ levels such that decreases in membrane PI(4,5)P_2_ levels decrease ENaC activity (26–30). ENaC activity is elevated in the *P2ry2*-knockout mouse (13, 22, 23). This is associated with decreased sodium excretion and elevated blood pressure, which is ameliorated by the ENaC blocker, amiloride. The increase in blood pressure observed in the *P2ry2*-knockout mouse has much in common with the elevated blood pressure found in humans harboring gain-of-function mutations in ENaC: both are sensitive to amiloride and associated with inappropriate Na^+^ retention and decreases in plasma K^+^, renin, and aldosterone concentrations (10, 11, 31). Moreover, knockout of *P2ry2* decreases sodium excretion and elevates blood pressure without affecting glomerular filtration rate (GFR) (10, 13, 14). Similarly, disrupting tubular secretion of ATP into the urine of the distal nephron abrogates pressure natriuresis, leading to a salt- and amiloride-sensitive increase in blood pressure (32). In comparison, activating *P2ry2* decreases blood pressure by increasing the fractional excretion of sodium and fluid, again without affecting GFR (11, 13). All of these observations are suggestive of a tubular mechanism of action.

However, due to imprecise experimental models, it has been difficult to directly test cause-and-effect relationships between changes in ENaC-dependent sodium excretion and *P2ry2*-dependent changes in blood pressure. To surmount this limitation, we created mice that have targeted knockout of *P2ry2* in renal principal cells and mice capable of pharmacogenetic activation of Gq signaling exclusively in renal principal cells. Blood pressure, sodium excretion, and ENaC activity were quantified in these models. Moreover, the consequences of stimulating *P2ry2* inhibition of ENaC in these mouse models were investigated in the context of deoxycorticosterone acetate–salt (DOCA-salt) hypertension (33, 34).

The current study tested the necessity and sufficiency of *P2ry2* signaling specifically in renal principal cells for effects on ENaC activity, sodium excretion, and blood pressure in principal cell–specific *P2ry2*-knockout mice and principal cell–specific Gq– designer receptors exclusively activated by designer drugs–knockin (Gq-DREADD–knockin) mice. Targeted deletion of *P2ry2* in principal cells abolished the effects that P2Y_2_ receptor agonism has on ENaC activity, renal sodium excretion, and blood pressure. Pharmacogenetic activation of Gq exclusively in principal cells decreased the activity of ENaC in renal tubules, causing a natriuresis capable of lowering elevated blood pressure. The current findings demonstrate that inhibition of ENaC activity in response to P2Y_2_ receptor–mediated Gq signaling lowered blood pressure by increasing renal sodium excretion. Thus, this purinoceptor is an emerging target capable of lowering elevated blood pressure, and its dysfunction may contribute to certain forms of hypertension.

## Results

### Activation of P2ry2 in principal cells stimulates sodium excretion.

**Figure 1:**
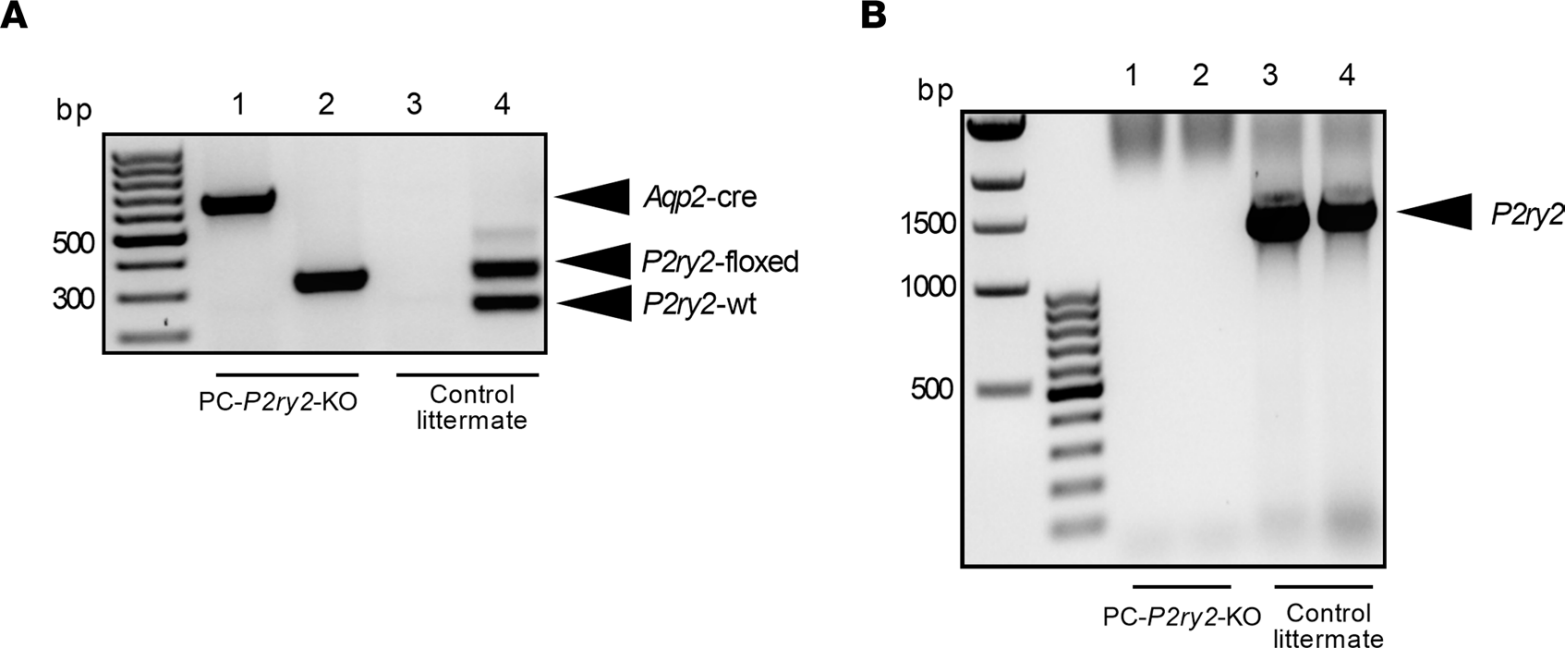
PC-*P2ry2*–KO mice. (**A**) Representative gel containing products from genotyping reactions for PC-*P2ry2*–KO mice (lanes 1 and 2) and littermate controls (lanes 3 and 4). Products (arrowheads) for the *Aqp2-Cre* transgene are shown in lanes 1 and 3. Products for the wild-type (wt) and floxed *P2ry2* transgene are shown in lanes 2 and 4. To increase visual clarity without changing content, contrast and brightness were adjusted, and the gray scale of this digital image was inverted. (**B**) Representative gel containing PCR products of exon 3 for the *P2ry2* gene from single-cell PCRs performed using genomic DNA from principal cells from 2 different PC-*P2ry2*–KO mice (lanes 1 and 2) and 2 different littermate controls (lanes 3 and 4). For presentation purposes, contrast and brightness were adjusted, and the image was inverted (black to white) to maximize clarity without changing content. bp, base pairs.

**Figure 2:**
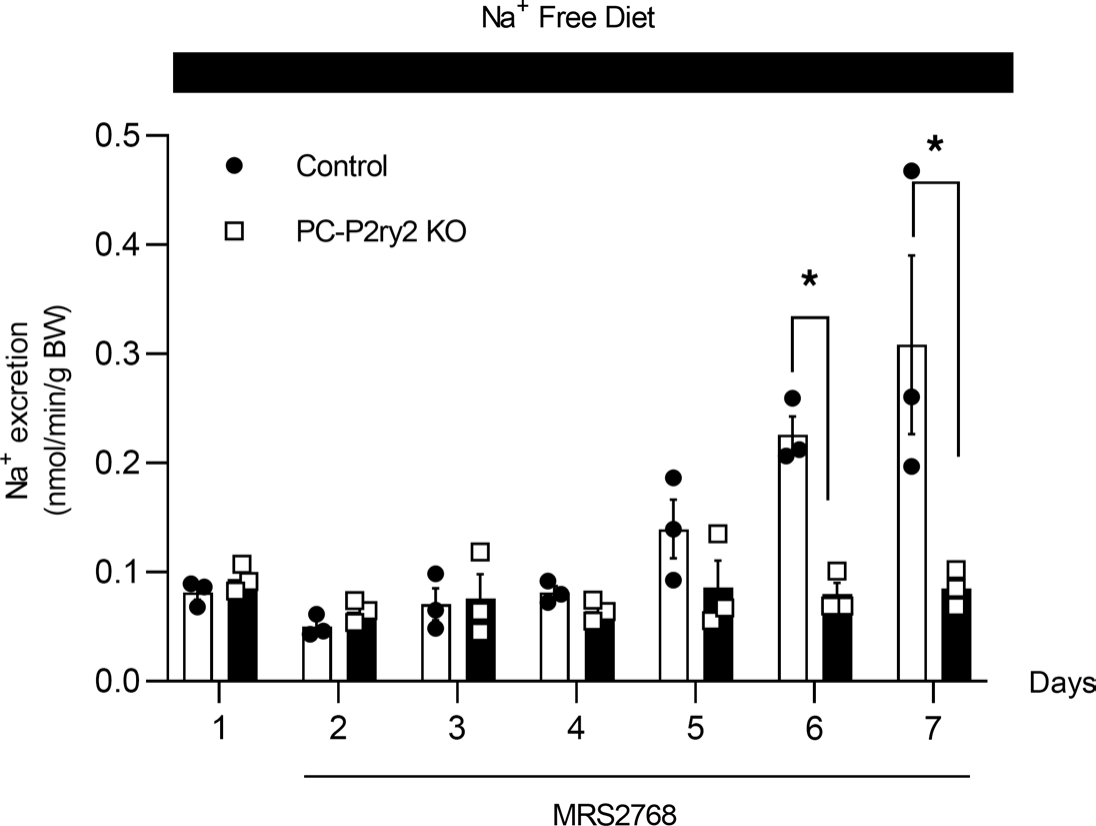
Deletion of *P2ry2* in principal cells abolishes stimulated increases in sodium excretion. Summary graph showing sodium excretion as a function of time for control littermates (white bars; *n* = 3 replications — 2 mice/replicate) and PC-*P2ry2*–KO mice (black bars, *n* = 3 replications — 2 mice/replicate) maintained on a sodium-free diet before and after treatment with the specific P2Y_2_ receptor agonist, MRS2768, for 6 consecutive days. MRS2768 was introduced daily via i.p. injections at 0.2 mg/kg in 100 μL of sterile water. Urinary sodium excretion (U_Na_V) was measured in 24-hour intervals. *P < 0.05 vs. KO mice via 2-way ANOVA and before addition of MRS2768.

Activating P2Y_2_ receptors in mice increases renal sodium excretion and decreases blood pressure (11). To determine the role played by P2Y_2_ receptors specifically in principal cells in this evoked sodium excretion, we compared changes in sodium excretion in response to selective activation of the P2Y_2_ receptor with MRS2768 in control littermates and principal cell–specific *P2ry2*-knockout (PC-*P2ry2*–KO) mice. For these experiments, we created and validated the PC-*P2ry2*–KO mouse as previously described (35). PC-*P2ry2*–KO mice were homozygous for the floxed *P2ry2* transgene and heterozygous for the aquaporin-2–Cre (Aqp2-Cre) transgene (Figure 1). Shown in Figure 1A are the products of a typical genotyping reaction for these lines. Littermate controls lacked the *Aqp2-Cre* transgene and/or the floxed *P2ry2* transgene. Furthermore, the fragment of exon 3 for the *P2ry2* gene from single-cell PCRs was verified using genomic DNA from principal cells from PC-*P2ry2*–KO mice and littermate controls. The presence of exon 3 was confirmed in DNA samples from all 6 principal cells from littermate controls and in 0 of 10 principal cells from PC-*P2ry2*–KO mice (Figure 1B). Sodium excretion was initially set to basal levels by maintaining mice with a sodium-free diet. As expected, and shown in Figure 2, targeted activation of P2Y_2_ receptors in wild-type control littermates (white bars) significantly increased renal sodium excretion from a basal value of 0.081 ± 0.007 to 0.226 ± 0.017 and 0.308 ± 0.081 nmol/min/g BW after 5 and 6 days of treatment with MRS2768, respectively. In PC-*P2ry2*–KO mice (black bars), activation of the P2Y_2_ receptor with MRS2768, in contrast, failed to increase sodium excretion, which was not different on days 5 (0.079 ± 0.011 nmol/min/g BW) and 6 (0.085 ± 0.017 nmol/min/g BW) of treatment compared to no treatment (0.093 ± 0.007 nmol/min/g BW). Resting sodium excretion in littermate controls and PC-*P2ry2*–KO mice was not different at steady state in the presence of a nominally sodium-free diet. However, evoked sodium excretion on days 5 and 6 of treatment with MRS2768 was significantly greater in littermate controls compared with PC-*P2ry2*–KO mice. These results demonstrate that the P2Y_2_ receptor specifically in renal principal cells is necessary for increases in renal sodium excretion in response to systemic activation of *P2ry2*.

### Activation of P2ry2 specifically in principal cells lowers blood pressure in the DOCA-salt model of hypertension.

**Figure 3:**
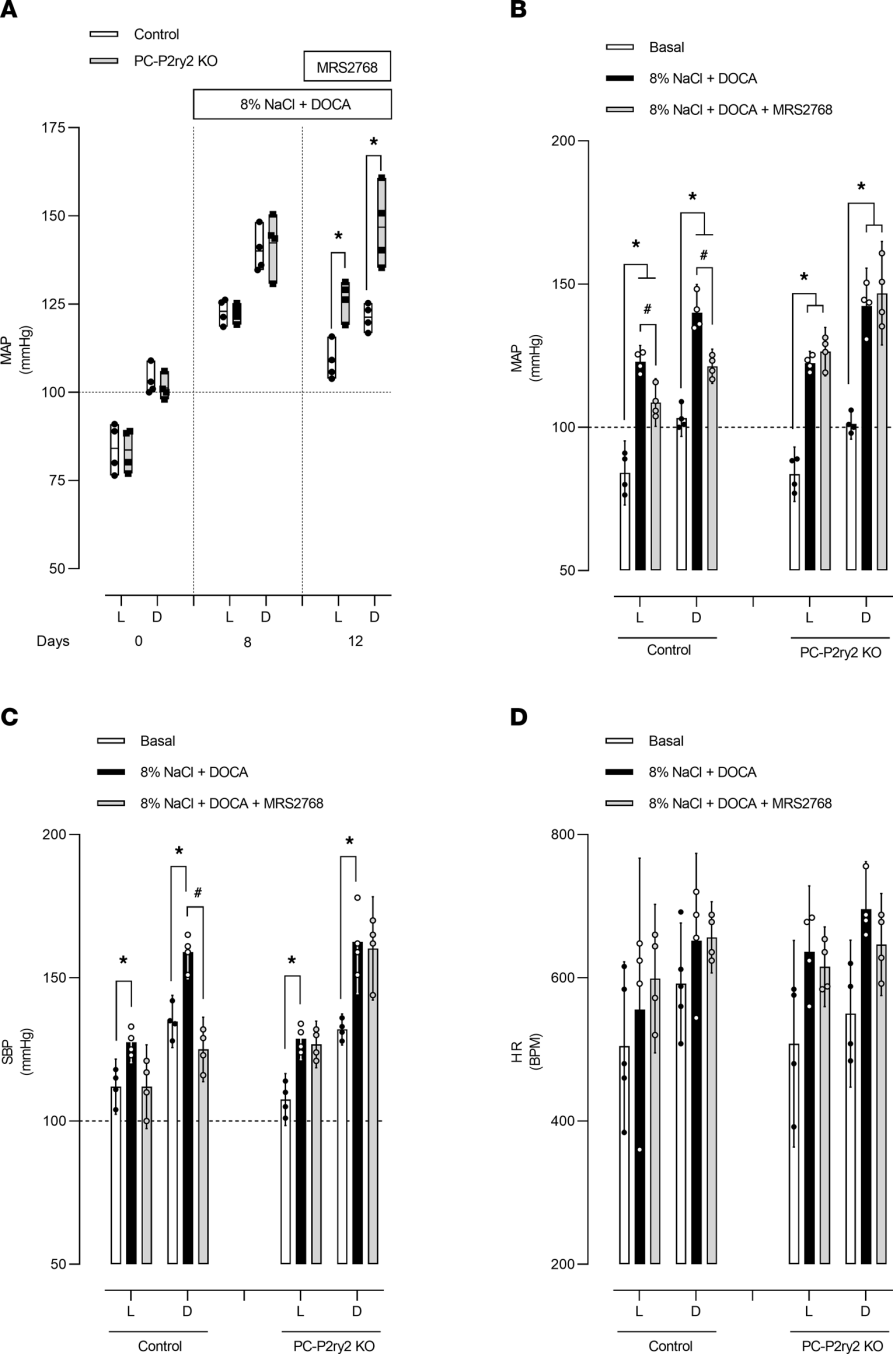
*P2ry2* expression in principal cells is necessary for regulation of blood pressure in the DOCA-salt model of hypertension. (**A**) Summary graph of MAP during the light (L) and dark (D) phases of the day for control littermates (white bars, *n* = 4 independent trials) and PC-*P2ry2*–KO mice (black bars, *n* = 4 independent trials). At all times mice had ad lib access to water. Mice were maintained with normal chow up to and through the beginning of the experiment (day 0). After this day, mice were implanted with DOCA pellets and maintained with an 8% NaCl diet to produce the DOCA-salt model of hypertension. MRS2768 (0.2 mg/kg) was introduced as daily (i.p.) injections on days 9–12. *P < 0.05 vs. control littermates with identical conditions. Summary histograms of MAP (**B**), SBP (**C**), and HR (**D**) for control littermates and PC-*P2ry2*–KO mice before (basal; white bars; day 0) and after DOCA-salt (black bars; day 8) and the addition of MRS2768 (gray bars; day 12). Data from experiments shown in **A**. *P < 0.05 within groups vs. basal (day 0) conditions, and ^#^*P* < 0.05 vs. DOCA-salt, via 2-way ANOVA.

To determine the necessity of P2Y_2_ receptors specifically in principal cells for P2Y_2_-sensitive decreases in blood pressure, we compared changes in mean arterial pressure (MAP), systolic blood pressure (SBP), and heart rate (HR) in a DOCA-salt model of hypertension in control littermates and PC-*P2ry2*–KO mice in response to treatment with the selective P2Y_2_ receptor agonist, MRS2768. As shown in Figure 3, A–D, with normal chow and ad lib water, MAP, SBP, and diastolic blood pressure (DBP, data not shown) during the light (L) and dark (D) phases of the day were not different for control littermates (L, 84.1 ± 3.5, D, 103.3 ± 2.0; L, 112.0 ± 3.0, D, 134.7 ± 2.9; L, 70.2 ± 5.5, D, 87.5 ± 3.6 mmHg, respectively) and PC-*P2ry2*–KO mice (L, 83.6 ± 2.9, D, 101.3 ± 1.7; L, 107.5 ± 2.8, D, 131.9 ± 1.8; L, 71.7 ± 4.7, D, 85.9 ± 2.7 mmHg, respectively). As expected, DOCA-salt significantly increased MAP, SBP, and DBP during both the light and dark phases of the day for control littermates (L, 122.9 ± 1.8, D, 140.1 ± 3.1; L, 127.5 ± 2.2, D, 159.0 ± 3.0; L, 120.6 ± 1.5, D, 130.6 ± 5.1 mmHg, respectively) and PC-*P2ry2*–KO mice (L, 122.4 ± 1.3, D, 142.4 ± 4.1; L, 128.7 ± 2.3, D, 162.5 ± 5.7; L, 119.2 ± 2.6, D, 132.3 ± 7.4 mmHg, respectively), but again MAP and SBP between these groups were not different. Subsequent treatment with MRS2768 (in the continued presence of DOCA-salt) significantly reduced MAP, SBP, and DBP in control littermates (L, 108.7 ± 2.6, D, 121.3 ± 1.9; L, 112.0 ± 4.6, D, 125.0 ± 3.6; L, 107.0 ± 3.2, D, 119.5 mmHg, respectively) but not PC-*P2ry2*–KO mice (L, 126.5 ± 2.6, D, 146.8 ± 5.7; L, 126.7 ± 2.5, D, 160.2 ± 5.6; L, 126.3± 4.1, D, 140.1 ± 6.5 mmHg, respectively). Moreover, MAP and SBP were both significantly greater in the PC-*P2ry2*–KO mice compared with control littermates in the presence of DOCA-salt plus MRS2768. This was true for both the light and dark phases of the day. As shown in Figure 3D, HR was not different between littermates and PC-*P2ry2*–KO mice under any condition, and treatment with DOCA-salt with or without MRS2768 did not affect HR.

### Activation of P2ry2 in principal cells decreases ENaC activity.

**Figure 4:**
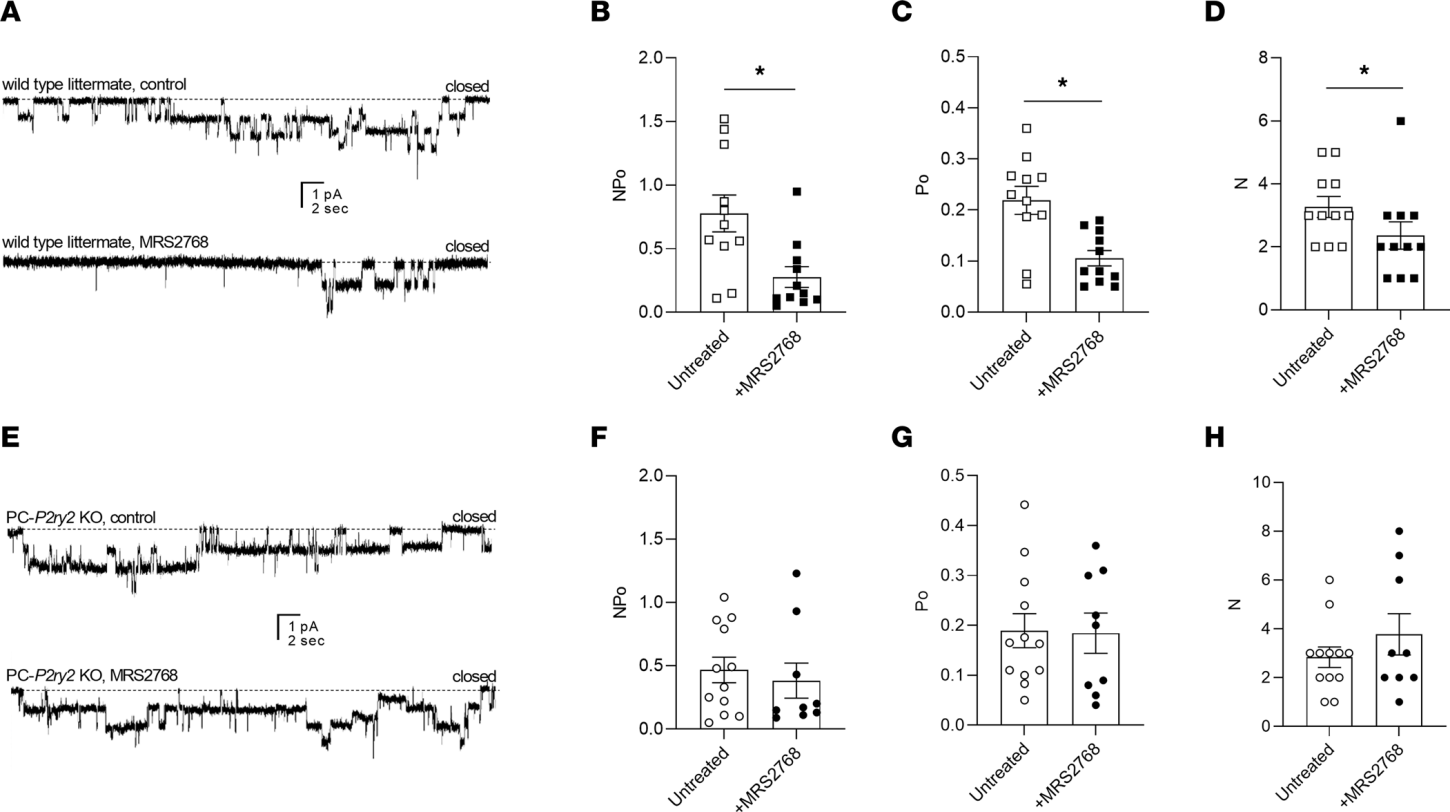
Expression of *P2ry2* is necessary for regulation of ENaC activity in principal cells. Representative gap-free current traces of ENaC in cell-attached patch-clamp recordings of the apical membranes of principal cells in isolated tubules from wild-type littermates (**A**) and PC-*P2ry2*–KO (**E**) mice in the absence (top) and presence (bottom) of pretreating isolated tubules with MRS2768 (5 μM, 30 minutes). Inward Na^+^ current is downward with dashed lines indicating the closed state. Summary graphs of ENaC activity (NPo), open probability (Po), and number (N) in the absence (white) and presence (black) of MRS2768 in tubules isolated from wild-type controls (**B**–**D**; boxes; *n* = 11, 11 patches from 2 male and 1 female mice for each condition) and PC-*P2ry2*–KO mice (**F**–**H**; circles, *n* = 12, 9 patches from 2 male and 1 female mice for each condition). These studies included mice of both sexes at approximately equal proportions. Summary data from experiments identical to those shown in **A** and **C**. *P < 0.05 vs. the absence of MRS2768, via 2-way ANOVA.

We tested next whether activation of P2Y_2_ receptors was capable of decreasing ENaC activity in principal cells in tubules from wild-type littermate controls versus PC-*P2ry2*–KO mice. Figure 4 shows representative current traces for ENaC in tubules isolated from control (Figure 4A) and PC-*P2ry2*–KO (Figure 4E) mice in the absence (top) and presence (bottom) of treatment with MRS2768. Figure 4, B–D and F–H, summarize all such results. Treatment with MRS2768 significantly decreased ENaC activity, open probability, and number in principal cells from control mice from 0.99 ± 0.19 to 0.28 ± 0.01, from 0.25 ± 0.03 to 0.11 ± 0.02, and from 3.62 ± 0.38 to 2.36 ± 0.43, respectively. In contrast, ENaC activity, open probability, and number were unaffected by MRS2768 in principal cells in tubules isolated from PC-*P2ry2*–KO mice (0.60 ± 0.16 vs. 0.77 ± 0.29, 0.204 ± 0.03 vs. 0.196 ± 0.03, and 2.8 ± 0.4 vs. 3.4 ± 0.7, respectively). The activity of ENaC in tubules from PC-*P2ry2*–KO mice in the absence of MRS2768 was not different than that in littermate controls. These results reveal the cellular and molecular mechanisms by which *P2ry2* agonism stimulates renal sodium excretion to decrease blood pressure.

### Sodium excretion is impaired in PC-P2ry2–KO mice.

**Figure 5:**
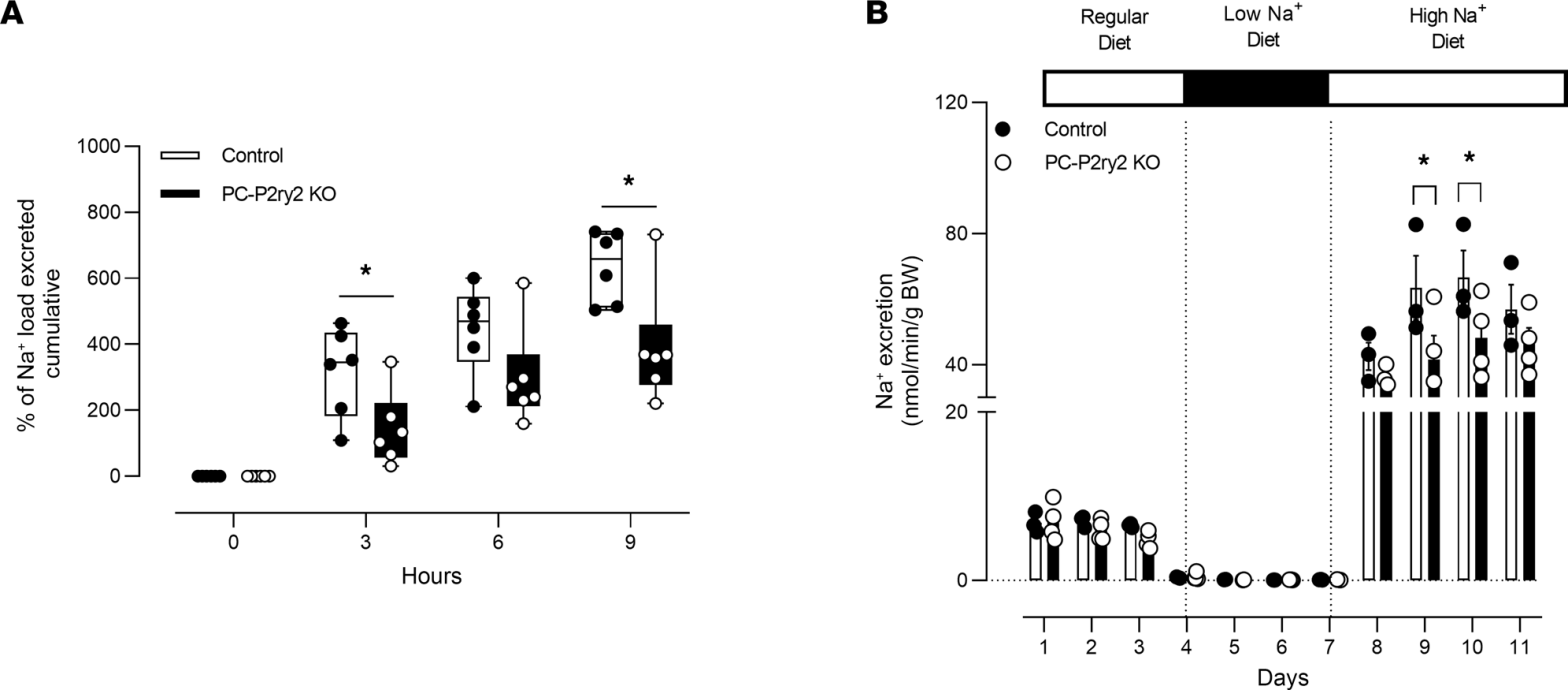
Sodium excretion is impaired in PC-*P2ry2*–KO mice. (**A**) Summary graph showing cumulative percentage excretion of a Na^+^ load (100 μL of 0.9% NaCl) as a function of time (measured every 3 hours) for control littermates (white circles, *n* = 6 independent trials) and PC-*P2ry2*–KO mice (black circles, *n* = 6 independent trials). Load introduced at time 0. Box plots show the interquartile range (box), median (line), and minimum and maximum (whiskers). *P < 0.05 vs. PC-*P2ry2*–KO. (**B**) Summary graph of 24 hours’ Na^+^ excretion for control littermate (white boxes; *n* = 3 replicates – 2 mice/replicate) and PC-*P2ry2*–KO (black circles; *n* = 3 replicates – 2 mice/replicate) mice maintained with normal, sodium-free, and high-sodium diets. *P < 0.05 vs. control littermates, via 2-way ANOVA.

To better understand the consequences of deleting *P2ry2* in principal cells in the living animal, we quantified further salt-dependent effects on U_Na_V in control littermates and PC-*P2ry2*–KO mice. Deletion of *P2ry2* in principal cells, as shown in Figure 5A, resulted in a decreased ability to excrete a sodium load. PC-*P2ry2*–KO (black box, white circle) mice compared with littermate controls (white box, black circle) provided a sodium load (100 μL, 0.9% saline) excreted significantly less sodium after 3 hours (143.5% ± 45.8% vs. 315.9% ± 54.8%) and 9 hours (391.0% ± 72.3% vs. 635.2% ± 44.4%).

Moreover, as shown in Figure 5B, PC-*P2ry2*–KO mice (black bar, white circle) initially retained sodium compared with control littermates (white bar, black circle) when transitioning from a state of low exogenous dietary sodium to excess dietary sodium. In the presence of regular chow and a nominally sodium-free diet, littermates and PC-*P2ry2*–KO mice had steady-state rates of sodium excretion of 6.83 ± 0.7 vs. 7.04 ± 1.1 and 0.08 ± 0.02 vs. 0.09 ± 0.02 nmol/min/g BW that were not different between groups but were reduced in both groups from normal chow to the sodium-free diet. Increasing dietary sodium to excess levels after maintenance with the sodium-free diet significantly increased sodium excretion in both littermate controls and PC-*P2ry2*–KO mice to peak values of 66.6 ± 8.2 vs. 48.2 ± 5.9 nmol/min/g BW after 3 days. While both increased, the magnitude of increase in PC-*P2ry2*–KO mice was significantly less than that in littermate controls. After day 3 on a high-sodium diet, sodium excretion in littermate controls and PC-*P2ry2*–KO mice trended toward each other to a common steady state that was not different between groups. These results demonstrate that the P2Y_2_ receptor in principal cells is necessary for normal sodium excretion when challenged with excessive dietary sodium, at least, initially until a new steady state is reached. As such, loss of the P2Y_2_ receptor in principal cells favors sodium retention in response to increased sodium intake.

### Pharmacogenetic activation of Gq in principal cells is sufficient to reduce ENaC activity.

**Figure 6:**
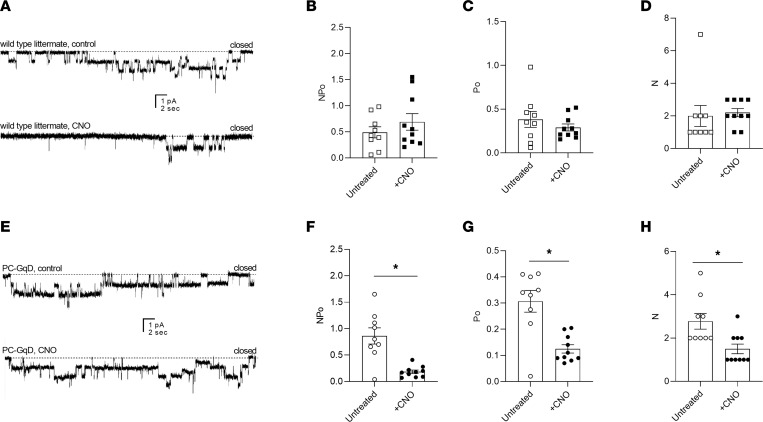
Targeted activation of Gq in principal cells is sufficient to decrease ENaC activity. Representative gap-free current traces of ENaC in cell-attached patch-clamp recordings of the apical membranes of principal cells in isolated tubules from wild-type littermates (**A**) and principal cell–specific Gq-DREADD–knockin (PC-GqD–knockin) (**E**) mice in the absence (top) and presence (bottom) of treatment with clozapine *N*-oxide (CNO) (5 μM; 30 minutes). Inward Na^+^ current is downward with dashed lines indicating the closed state. Summary graphs of ENaC activity (NPo), open probability (Po), and number (N) in the absence (white) and presence (black) of CNO in tubules isolated from wild-type controls (**B**–**D**; boxes; *n* = 9, 10 patches from 2 male and 1 female mice for each condition) and PC-*P2ry2*–KO mice (**F**–**H**; circles, *n* = 9, 9 patches from 2 male and 1 female mice for each condition). These studies included mice of both sexes at approximately equal proportions. Summary data from experiments identical to those shown in **A** and **C**. *P < 0.05 vs. the absence of CNO, via 2-way ANOVA.

Stimulating P2Y_2_ receptors in principal cells activates Gq signaling. Because P2Y_2_ receptors are necessary for normal ENaC activity, dependent sodium excretion, and regulation of blood pressure (see above), we wondered whether stimulating Gq signaling exclusively in principal cells would be sufficient to decrease ENaC activity, promote sodium excretion, and consequently, decrease elevated blood pressure. To test this, we used pharmacogenetics to selectively activate Gq in principal cells. Figure 6 shows representative current traces for ENaC in tubules isolated from control (Figure 6A) and PC-GqD (Figure 6E) mice in the absence (top) and presence (bottom) of CNO. Figure 6, B–D and F–H, summarize all such results. ENaC activity, open probability, and number of control littermates in the absence and presence of CNO were not different at 0.49 ± 0.11 and 0.69 ± 0.16, 0.38 ± 0.08 and 0.29 ± 0.04, and 1.9 ± 0.59 and 2.2 ± 0.25, respectively. In contrast, treatment with CNO markedly decreased ENaC activity in principal cells from PC-GqD mice from 0.86 ± 0.15 to 0.18 ± 0.33. In the presence of CNO, ENaC activity was substantially lower in PC-GqD mice compared to littermate controls. Thus, selective activation of Gq in principal cells is sufficient to decrease ENaC activity.

### Activating Gq in principal cells increases sodium excretion.

**Figure 7:**
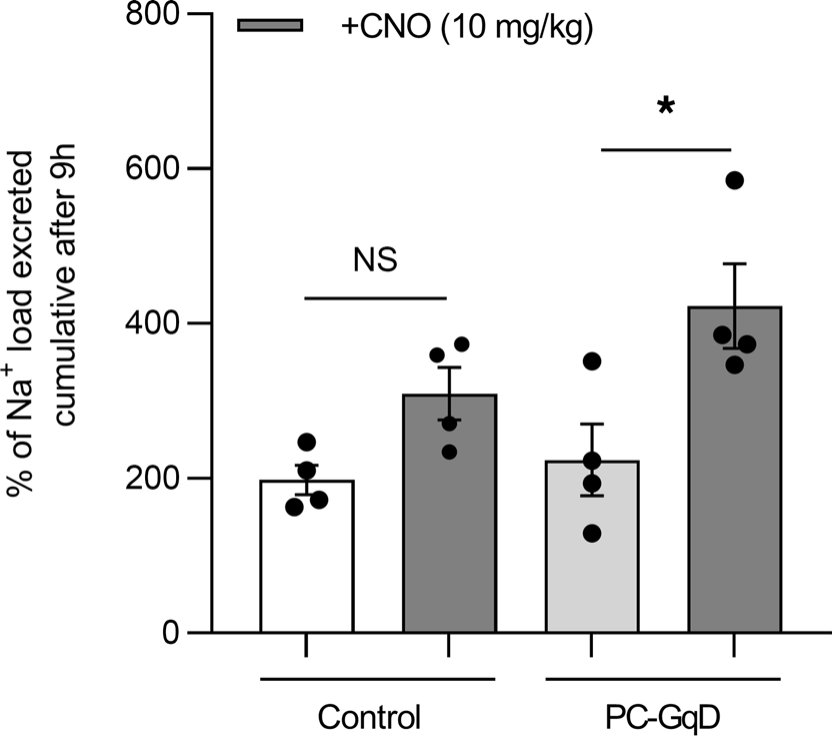
Targeted activation of Gq in principal cells is sufficient to increase sodium excretion. Summary graph of cumulative percentage excretion of a Na^+^ load (100 μL of 0.9% NaCl) after 9 hours for control littermates (white) and PC-GqD–knockin (light gray) mice in the absence (open bars) and presence of CNO (dark gray; 10 mg/kg introduced with the Na^+^ load). For each condition, *n* = 5 independent trials. *P < 0.05 vs. the absence of CNO, via 2-way ANOVA.

If selective activation of Gq in principal cells decreases ENaC activity, then this should be sufficient to increase sodium excretion. As shown in Figure 7, the cumulative excretion of a sodium load (100 μL, 0.9% saline) after 9 hours was not different in the absence of CNO in PC-GqD mice (224.0% ± 46.6%) compared with littermate controls (198.0% ± 19.3%). However, in the presence of CNO, PC-GqD mice (422.3% ± 54.7%) but not littermate controls (309.4% ± 33.7%) excreted a greater amount of the sodium load after 9 hours compared with untreated mice within the same group. A 2-way ANOVA showed that the strain and interaction effects were not statistically significant, while the treatment effect was statistically significant (*P* = 0.0026).

### Activating Gq in principal cells is sufficient to reduce blood pressure.

**Figure 8:**
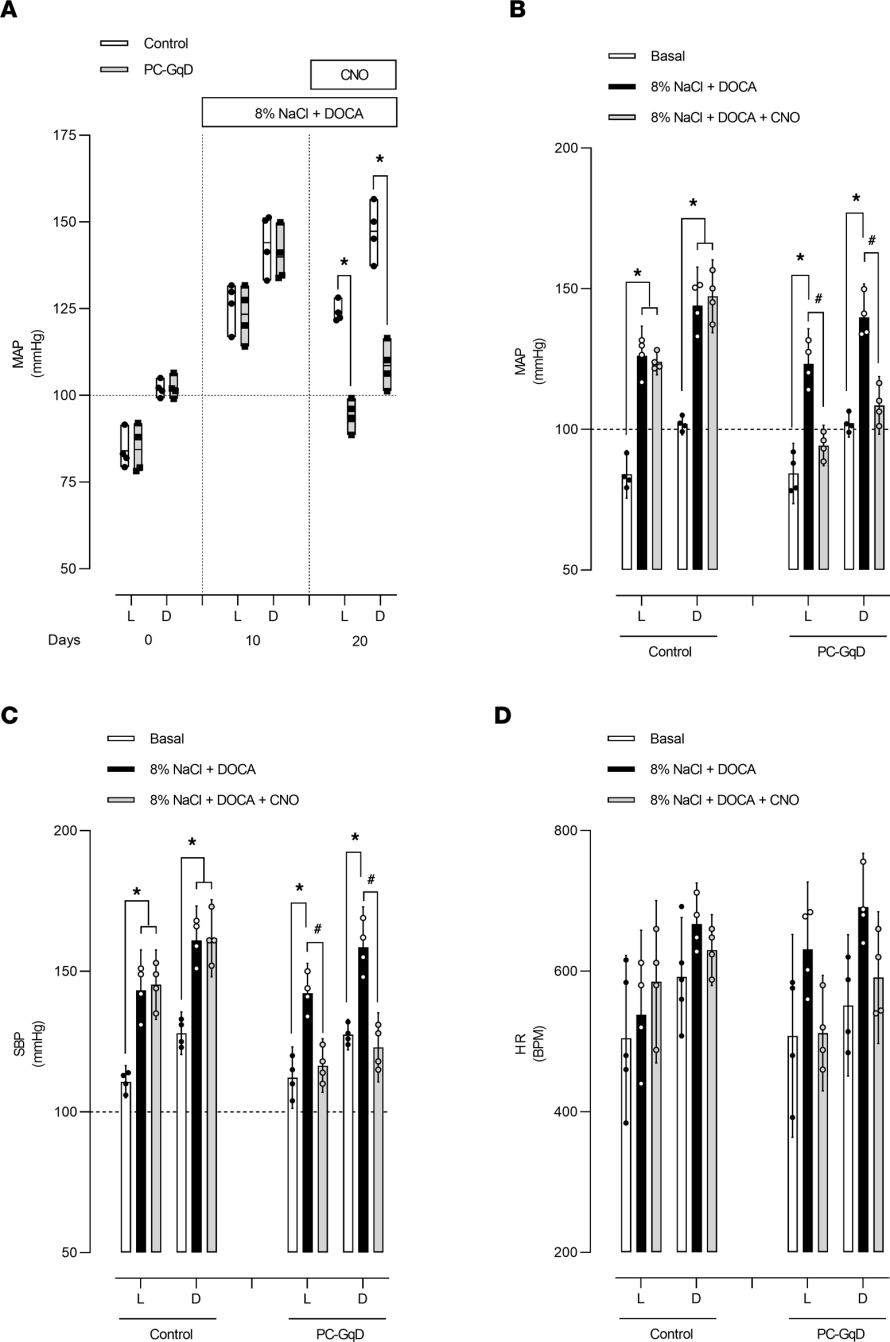
Targeted activation of Gq in principal cells is sufficient to decrease blood pressure in the DOCA-salt model of hypertension. (**A**) Summary graph of MAP during the light (L) and dark (D) phases of the day for control littermates (white bars, *n* = 4 independent trials) and PC-GqD–knockin (black bars, *n* = 4 independent trials) mice. At all times mice had ad lib access to water. Mice were maintained with normal chow up to and through the beginning of the experiment (day 0). After this day, mice were implanted with DOCA pellets and maintained with an 8% NaCl diet to produce the DOCA-salt model of hypertension. CNO (10 mg/kg) was introduced via daily (i.p.) injections on days 15–20. *P < 0.05 vs. control littermates under identical conditions. Summary histograms of MAP (**B**), SBP (**C**), and HR (**D**) for control littermates and PC-GqD–knockin mice before (basal; white bars; day 0) and after DOCA-salt (black bars; day 10) and the addition of CNO (gray bars; day 20). Data from experiments shown in **A**. *P < 0.05 within groups vs. basal (day 0) conditions, and ^#^*P* < 0.05 vs. DOCA-salt, via 2-way ANOVA.

If targeted activation of Gq in principal cells decreases ENaC activity to promote a natriuresis, then such activation may be sufficient to decrease elevations in blood pressure. To test this possibility, we used pharmacogenetics to selectively activate Gq in principal cells in a DOCA-salt model of hypertension in PC-GqD mice and littermate controls. As shown in Figure 8, A–C, MAP, SBP, and DBP (data not shown) with normal chow were not different between PC-GqD mice (L, 84.3 ± 3.4, D, 102.2 ± 1.6; L, 112.2 ± 3.4, D, 127.5 ± 1.7; L, 70.4 ± 4.4, D, 89.6 ± 2.4 mmHg, respectively) and littermate controls (L, 84.0 ± 2.6, D, 101.9 ± 1.2; L, 110.7 ± 1.8, D, 128.0 ± 2.4; L, 40.7 ± 4.2, D, 62.0 ± 3.1 mmHg, respectively). As expected, DOCA-salt significantly increased MAP, SBP, and DBP during both the light and dark phases of the day in both PC-GqD mice (L, 123.4 ± 3.9, D, 139.9 ± 3.7; L, 142.2 ± 3.3, D, 158.5 ± 4.5; L, 113.9 ± 3.9, D, 130.6 ± 3.8 mmHg, respectively) and littermate controls (L, 126.2 ± 3.3, D, 144.1 ± 4.3; L, 143.2 ± 4.5, D, 161.0 ± 3.8; L, 117.7 ± 2.4, D, 135.6 ± 4.8 mmHg, respectively). With DOCA-salt, MAP and SBP were not different between littermate controls and PC-GqD mice. Subsequent treatment, though, with CNO (in the continued presence of DOCA-salt) significantly reduced MAP in PC-GqD mice (L, 94.3 ± 2.3, D, 108.6 ± 3.2; L, 116.5 ± 2.9, D, 123.0 ± 3.8 mmHg) but not littermate controls (L, 124.1 ± 1.5, D, 147.3 ± 4.1; L, 145.2 ± 3.8, D, 161.7 ± 4.3 mmHg). Both MAP and SBP, moreover, were significantly lower in PC-GqD mice compared with control littermates in the presence of DOCA-salt plus CNO. This was true for both the light and dark phases of the day. As shown in Figure 8D, HR was not different between littermates and PC-GqD mice under any condition, and treatment with DOCA-salt with or without CNO failed to affect HR. These results are consistent with activation of Gq exclusively in principal cells being sufficient to decrease elevated blood pressure in the DOCA-salt model of hypertension, with Gq-dependent decreases in ENaC activity and subsequent increases in sodium excretion being the molecular and cellular mechanisms underpinning this decrease in blood pressure.

## Discussion

The current studies demonstrate that activating *P2ry2* and Gq signaling in renal principal cells is sufficient to decrease the activity of ENaC, evoking a natriuresis capable of lowering elevated blood pressure. Moreover, these results demonstrate that *P2ry2* specifically in principal cells is necessary for P2Y_2_ receptor agonism to increase Na^+^ excretion and decrease elevated blood pressure. As such, these results highlight the central importance of regulation of ENaC by *P2ry2* signaling in renal principal cells to the control of blood pressure. They emphasize that this signaling pathway, while possibly functioning in parallel with actions on vascular smooth muscle, is powerful enough alone to influence blood pressure.

This is the second report of *P2ry2* agonism evoking a natriuresis capable of decreasing blood pressure. The first study, by Rieg and colleagues (11), showed that *P2ry2* agonism with INS45973 dose-dependently increased renal sodium excretion and significantly decreased blood pressure. These effects were retained in endothelial nitric oxide synthase–KO mice but were absent in the global *P2ry2*-KO mouse. Effects on excretion of sodium and fluid as well as blood pressure in this earlier study were independent of changes in GFR and retained when normalized to filtration rate. Such observations are consistent with a renal mechanism involving an increase in sodium excretion in response to *P2ry2* agonism driving decreases in blood pressure. The current study is in agreement with such a mechanism. We report here that the chemically distinct *P2ry2* agonist, MRS2768, drives a natriuresis in mice maintained with a sodium-free diet and decreases blood pressure in the DOCA-salt model of hypertension. Both effects are absent in the PC-*P2ry2*–KO mouse. These findings further highlight the specific site of activity and demonstrate that *P2ry2* in renal principal cells plays a crucial role in regulating sodium excretion and blood pressure. Results in Figure 4 show that *P2ry2* agonism decreases the activity of ENaC in renal principal cells and that this effect is absent in PC-*P2ry2*–KO mice. This observation identifies inhibition of ENaC as the major cellular mechanism whereby *P2ry2* signaling decreases blood pressure. In this sense, then, *P2ry2* regulation of blood pressure shares parallels with how the potassium-sparing diuretics, amiloride and triamterene, affect renal sodium and fluid excretion to decrease blood pressure. Both amiloride and triamterene are ENaC blockers (36). While the current studies do not exclude possible contribution from effects on the vasculature, they do demonstrate that *P2ry2* signaling to ENaC in principal cells of the distal nephron is a key signaling pathway for the control of blood pressure. Other hypotheses of the regulation of ENaC by P2Y2 receptor could be more physiologically relevant during acute changes.

Here we demonstrated that MRS2768 decreases the blood pressure in the DOCA-salt model of hypertension. Such decrease in blood pressure is probably due to MRS2768’s effect on the *P2ry2* expressed in the kidneys as no effect was observed in the PC-*P2ry2*–KO mouse (Figure 3). However, we cannot exclude a possible effect on the vasculature. The participation of *P2ry2* in vasodilation has been investigated in several studies. Previous studies have demonstrated that the global *P2ry2*-KO model develops salt-resistant hypertension (37), impaired ATP-evoked relaxation in aorta (38), and greater infarct size and impaired heart function in a myocardial infarction model, as in vivo treatment using MRS2768 not only protects the heart from ischemic damage (39, 40) but also prevents vascular calcification (41, 42). Our data demonstrated that in isolated tubules MRS2768 has a direct effect in the P2Y2 receptor present in the principal cell, and upon its activation, ENaC activity is reduced. In vivo, though, the response to this agonist was observed after 72 hours. Importantly, the increase in sodium excretion was observed in a condition of low-sodium diet, in which ENaC is fully active in order to retain the sodium balance. One possible explanation for such observation may be related to liver metabolizing and degradation of the compound before reaching the receptor target. These data verify that the compound is specific for the P2Y_2_ receptor. However, we cannot exclude its actions on other sites such as the vascular system.

Moreover, deleting *P2ry2* specifically in principal cells had no effect on salt balance when mice were fed a low-salt diet or a regular diet. Results in Figure 5B show that when mice were fed a high-salt diet, they show a marked increase in sodium excretion; however, PC-*P2ry2*–KO mice show a small but statistically significant difference when compared with littermate controls. In addition, results in Figure 3 show that deletion of *P2ry2* deletion specifically in principal cells did not alter blood pressure at baseline or when mice were maintained with high salt and DOCA. This likely results from regulation of blood pressure being multifactorial as many other systems remain intact in the PC-*P2ry2*–KO mice.

The current findings that stimulation of Gq signaling solely in renal principal cells is sufficient to decrease ENaC activity, causing a natriuresis capable of decreasing blood pressure, also agree that this pathway is important to the control blood pressure. That *P2ry2*/Gq signaling is necessary and sufficient for the inhibition of ENaC is consistent with several earlier studies. Using pharmacology and patch-clamping of principal cells in freshly isolated renal tubules from the *P2ry2*-KO mouse, we previously demonstrated that ATP and UTP decrease the activity of ENaC via the P2Y_2_ receptor (22). Activation of *P2ry2* decreases ENaC activity by stimulating PLC through Gq signaling (22), as PI(4,5)P_2_ present at the apical membrane is catabolized by PLC activation. In this way, to maintain appropriate ENaC gating and activity, PI(4,5)P_2_ is required. ENaC binds PI(4,5)P_2_ using cytosolic NH_2_ and COOH terminal domains (30, 43, 44) rich in conserved basic amino acids. The binding of PI(4,5)P_2_ to these domains relieves the channel from self-inhibition by sequestering away a negative gating element (43).

That urinary ATP acts in a paracrine manner to decrease ENaC activity is by now well supported (24, 45). The key source(s) of this urinary ATP and what causes ATP to be released into the urine are also beginning to emerge. One important source of urinary ATP with respect to paracrine regulation of ENaC is intercalated cells (13, 24). The distal tubule is mainly constituted by 2 cell types: principal cells and intercalated cells. In response to increases in urinary flow, ATP is released from intercalated cells (46, 47). Such releases of ATP act as a surrogate for increased blood pressure and/or increases in sodium consumption, empowering a feedback response to counter increases in blood pressure by increasing sodium excretion (48).

Lack of this feedback response was used to explain, at least in part, why blood pressure is elevated in global *P2ry2*-KO mice (10). Interestingly, increases in blood pressure worsen in the *P2ry2*-KO mouse when they are maintained with high salt and DOCA (10).

The global *P2ry2*-KO mice have multiple alterations in their handling of salt and water, which include suppressed plasma renin and aldosterone concentrations, lower renal expression of the aldosterone-induced epithelial sodium channel α-ENaC, greater medullary expression of the Na-K-2Cl cotransporter NKCC2, and greater furosemide-sensitive Na^+^ reabsorption in association with greater renal medullary expression of Aqp2 and vasopressin-dependent renal cAMP formation and water reabsorption, despite similar vasopressin levels compared to wild-type (10). Such alterations lead to salt-resistant arterial hypertension that is associated with an inverted relationship between salt intake and HR. To verify the renal role of *P2ry2* in regulating blood pressure, we used the PC-specific *P2ry2* animal model. We verified that deletion of *P2ry2* in principal cells abolishes increases in sodium excretion in response to stimulation of P2Y_2_ receptors and compromises the normal ability to excrete a sodium load. Consequently, principal cell–specific KO of *P2ry2* prevents decreases in blood pressure in response to P2Y_2_ receptor stimulation in the DOCA-salt model of hypertension. These findings demonstrate that the kidneys play a major role in decreasing blood pressure in response to P2Y_2_ receptor activation.

Importantly, a clear difference between the global *P2ry2*-KO mice, salt resistant, and the principal cell–specific KO of *P2ry2*, salt sensitive, demonstrated the key role of *P2ry2* in controlling blood pressure. The main reasons the global *P2ry2*-KO mice have a salt-resistant hypertension have been previously explored (10). The salt-sensitive hypertension observed in the principal cell–specific KO of *P2ry2* could be explained by the fact that this receptor was ablated mainly in the kidneys. We can mainly speculate that such differences are based on the animal model. We clearly demonstrated that principal cell–specific KO of *P2ry2* showed impaired Na^+^ excretion and salt sensitivity. When deleted using Aqp2, either constitutive or inducible (49, 50), the mice appear normal at baseline and show salt wasting only upon challenge. In contrast, when mineralocorticoid receptor is deleted along the entire nephron, ENaC activity is dramatically low, and the mice waste salt at baseline (51, 52). Similar findings have been reported when ENaC itself is deleted either along the collecting duct or the entire nephron. Thus, the phenotypic difference between whole body and principal cell–specific knockout could be related to ENaC expressed along the late distal collecting tubule and the early connecting tubule (CNT), segments that do not express Aqp2. Our study focused mainly on the regulation of ENaC activity by *P2ry2* receptors. Reactive oxygen species (ROS) are produced in the kidney in response to aldosterone and sodium balance. High-salt diet induces kidney injury by increasing oxidative stress. Recently, our group and others have demonstrated that NADPH oxidase activator 1–dependent NADPH oxidase 1 (NOXA1/NOX1) signaling plays a role in angiotensin II–dependent (ANG II–dependent) activation of ENaC. ANG II activation of renal NOXA1/NOX1-dependent ROS enhances tubular ENaC expression and Na^+^ reabsorption, leading to increased blood pressure (53, 54). These observations are important as they suggest an important role played by ROS leading to ENaC activity and Na^+^ retention. That extracellular ATP can modulate redox biology has been previously demonstrated (55). Extensive details on the mechanism by which ATP induces ROS generation and its association with purinergic receptors can be found elsewhere (56). Not only that, intracellular Ca^2+^ and ROS generation can activate signaling pathways modulated by purinergic receptors, leading to activation of protein kinase C, MAP kinase members, and phospholipase A2, which may reflect on ENaC expression levels. Such mechanism could be associated with the impaired Na^+^ excretion and salt-sensitive hypertension observed in the principal cell–specific knockout of *P2ry2*.

Using potentially novel principal cell–specific *P2ry2*-knockout mice and principal cell–specific Gq-DREADD–knockin mice, the current study tests the necessity and sufficiency of *P2ry2* signaling specifically in renal principal cells for effects on ENaC, sodium excretion, and blood pressure. As such, these studies reveal the contribution made by the kidneys to *P2ry2* control of blood pressure and elaborate the cellular and molecular mechanisms by which P2Y_2_ receptor activation in the kidneys contributes to the control of blood pressure. Conditional deletion of *P2ry2* in principal cells abolishes the effects that P2Y_2_ receptor agonism has on ENaC activity, renal sodium excretion, and blood pressure. Pharmacogenetic activation of Gq exclusively in principal cells decreases the activity of ENaC in renal tubules, promoting a natriuresis that lowers elevated blood pressure.

In conclusion, the current findings are consistent with the kidneys playing a major role in decreasing blood pressure in response to P2Y_2_ receptor activation, and the inhibition of ENaC activity in response to P2Y_2_ receptor–mediated Gq signaling lowers blood pressure by increasing renal sodium excretion.

## Methods

### Animal care and use.

All animal use and welfare adhered to the NIH *Guide for the Care and Use of Laboratory Animals* (National Academies Press, 2011) following protocols reviewed and approved by the Institutional Animal Care and Use Committee of the University of Texas Health Science Center at San Antonio. Mice were housed and cared for in the Laboratory Animal Resources Facility at the University of Texas Health Science Center at San Antonio. This facility is fully accredited by the Association for Assessment and Accreditation of Laboratory Animal Care and licensed by the US Department of Agriculture.

Healthy young adult (2–3 months old, ~23 g BW) male and female mice, in approximately equal proportions, were used for experiments as a source of CNT/cortical collecting duct for patch-clamp analysis of ENaC activity, for excretion studies, and for measurement of blood pressure with telemetry (see below). Within a genotype, assignment to the experimental or control group was random. Before, during, and after experiments, mice were maintained on a normal 12-hour light/12-hour dark cycle at room temperature with free access to water and standard chow (TD.7012 and/or TD.8656; ENVIGO) and were housed socially with littermates and peers.

For some experiments, mice were maintained with a Na^+^-free diet (≤0.02% Na^+^; TD.90228; ENVIGO) and/or a high-Na^+^ diet (4% NaCl; TD.92034; ENVIGO). In another set of experiments, a standard DOCA-salt model of hypertension was used (33, 34). For this, mice were implanted with subcutaneous DOCA pellets (50 mg/pellet, 21-day release: catalog M-121, Innovative Research of America) and maintained with a high-Na^+^ diet (8% NaCl, TD.92012; ENVIGO).

All acute experimental maneuvers, including intraperitoneal injection of CNO (catalog 6329; Tocris), intraperitoneal injection of MRS2768 (uridine-5′-tetraphosphate δ-phenyl ester; catalog 3884; Tocris), and intraperitoneal introduction of a sodium load (100 μL 0.9% saline), were performed within a constant time window (midmorning) and setting (the laboratory).

### Creation and validation of the principal cell–specific P2ry2-knockout mouse.

PC-*P2ry2*–KO mice were homozygous for the floxed *P2ry2* transgene and heterozygous for the *Aqp2-Cre* transgene. Littermate controls lacked the *Aqp2-Cre* transgene and/or the floxed *P2ry2* transgene. The *Aqp2-Cre* transgene was transmitted only through the female line. The floxed *P2ry2* transgene contained *loxP* sites in the untranslated regions before and after exon 3. These sites enabled Cre-recombinase–mediated deletion of the *P2ry2* exon 3 coding region. Floxed *P2ry2* mice were created by C. Seye (Indiana University School of Medicine, Indianapolis, Indiana, USA) and have been described previously (35). The B6.Cg-Tg (Aqp2-Cre) 1Dek/J mouse was from D. Kohan (University of Utah Health Science Center, Salt Lake City, Utah, USA) and has been described previously (57, 58). Principal cell–specific deletion of *P2ry2* did not result in any noticeable developmental or behavioral abnormality or pathology in unstressed animals.

For genotyping reactions, as presented in Figure 1A, the floxed *P2ry2* transgene was identified using the forward 5′-TGACGACTCAACACGGACAG-3′ and reverse 5′-TCCCAACTCACAGCTACAAATG-3′ PCR primers, which produced expected 329 bp products for the floxed allele and 246 bp products for the wild-type allele. The *Aqp2-Cre* transgene was identified with the forward 5′-CTCTGCAGGAACTGGTGCTGG-3′ and reverse 5′-GCGAACATCTTCAGGTTCTGCGG-3′ PCR primers, producing an expected 673 bp product.

Single-cell PCR was performed using genomic DNA from principal cells in freshly isolated tubules to assess the efficiency of removal of exon 3 upon recombination to confirm targeted deletion of *P2ry2*. DNA was isolated from single principal cells that had hallmark ENaC expression as confirmed by patch-clamp electrophysiology (described below) using the patch pipette. Isolated DNA was purified and made ready for PCR using the Single Cell Lysis Kit (Thermo Fisher Scientific). In PCRs, the forward 5′-CAGGCTACCCAAGCAAACCTTCAGCA-3′ and reverse 5′-CCTGCCAAGTGAGAGCTGTTGATCC-3′ primers, producing an expected 1,492 bp product, were used to discriminate between the presence and absence of exon 3. Representative results for these single-cell PCRs are shown in Figure 1B. A total of 6 distinct principal cells from 3 distinct littermate controls and 10 distinct principal cells from 3 distinct PC-*P2ry2*–KO mice were tested. The presence of exon 3 was confirmed in DNA samples from all 6 principal cells from littermate controls and in 0 of 10 principal cells from PC-*P2ry2*–KO mice. These proportions are significantly different as tested with a 2-sample *z* test.

### Creation and validation of the principal cell–specific Gq-DREADD mouse.

Creation and validation of the PC-GqD mouse have been described previously (12). PC-GqD mice were homozygous for the *CAG-LSL-Gq-DREADD* transgene and heterozygous for the *Aqp2-Cre* transgene, with the latter being passed only through the female line. Littermate controls lacked the *Aqp2-Cre* and/or *CAG-LSL-Gq-DREADD* transgene.

### Split-open tubule preparation and single-channel patch-clamp electrophysiology.

Renal tubules were isolated, split open, and prepared for patch-clamp electrophysiology as previously described (12, 38, 39). In brief, kidneys were sectioned transversely, and segments of the CNT and cortical collecting duct were manually micro-dissected with forceps and adhered to a glass cover-chip coated with 0.01% poly-l-lysine. Chips then were transferred to an inverted microscope, where the top layer of the CNT/cortical collecting duct was split open with sharpened pipettes. Gap-free, single-channel, voltage-clamp electrophysiology in the cell-attached configuration was then performed using standard procedures on the luminal plasma membranes of principal cells in split-open tubules (12, 38, 39). For these experiments, the bath solution contained (in mM): 150 NaCl, 5 KCl, 1 Cacl_2_, 2 MgCl_2_, 5 glucose, and 10 HEPES (pH 7.4). The pipette solution contained (in mM): 140 LiCl, 2 MgCl_2_, and 10 HEPES (pH 7.4). A current-voltage correlation was established for ENaC, which had a conductance of 4–5 pS. The pipette contained 140 mM NaCl, and pipette resistance was approximately 7–10 mΩ. In these cell-attached voltage-clamp studies, currents were low-pass-filtered at 100 Hz. Channel activity (open probability multiplied by channel number, *N*) was calculated as previously described (12, 38, 39). Channel activity, defined as NPo, was calculated using the equation NPo = Σ(t_1_ + 2t_2_ + … it_i_), where t_i_ is the fractional open time spent at each of the observed current levels. Open probability was estimated by normalizing NPo for the observed number of channels within a patch. The error that is associated with this estimation of open probability increases as patches contain more channels and as open probability approaches either 0 or unity. Only patches that contained 5 channels or fewer were used to estimate open probability. Single-channel conductance was calculated by recording at least 3 holding potentials (59, 60).

### Metabolic cage experiments.

Metabolic cage experiments quantifying Na^+^ excretion closely followed previously published protocols (12, 38, 39). In brief, age- and weight-matched mice were housed in metabolic cages (2 mice/cage: Techniplast). Urinary Na^+^ concentration and urine volume were quantified every 24 hours over the course of the experiment, with daily Na^+^ excretion (U_Na_V) reported. Urinary Na^+^ concentration was quantified with a flame photometer (model PFP7, Jenway).

In another set of experiments, mice were provided a sodium load (100 μL of 0.9% saline, i.p.). The cumulative percentage of the sodium load excreted then was quantified over the next 9 hours by collecting urine and measuring Na^+^ concentration and volume.

### Measurement of blood pressure with telemetry.

Standard techniques for mice were used to place and use radio telemeters to measure blood pressure (40, 41). In brief, a catheter was inserted into the left carotid artery of anesthetized (isoflurane) mice and advanced to place the blood pressure sensor in the aorta. The radio telemetry device (TA11PA-C10, Data Science International) was embedded subcutaneously in the abdominal region. Each animal received standard postoperative care to include treatment with penicillin G procaine (3,000 units, Aspen Veterinary Resources Ltd) and buprenorphine (0.09 mg/kg, MWI Animal Health). Antibiotic treatment continued for 5 days. Analgesic was provided every 8–12 hours for 3 days following surgery.

SBP, DBP, and HR were continuously recorded (at 1,000 Hz) over the course of these experiments in fully conscious and freely behaving instrumented mice for 1 minute 10 times an hour. Telemetric recording of blood pressure and HR was acquired with a PhysioTel System (Data Science International). Telemetry data were collected and analyzed using Spike2 (version 6.06, Cambridge Electronic Design). MAP was calculated as standard as (1/3 × SBP) + (2/3 × DBP). For individual mice, blood pressure measurements were the average of all data points on a given day split into the light and dark phase of that day.

Two protocols were used to collect and report blood pressure data. For PC-*P2ry2*–KO mice, telemeters were implanted on day –3, with mice maintained on standard chow prior to and during recovery until day 0. Starting blood pressures were reported on day 0. On day 1, DOCA pellets were implanted, and mice switched to the 8% NaCl diet for the next 12 days. The effects of DOCA-salt on blood pressure were reported on day 8. Daily MRS2768 injections began on day 9 through day 12, with the effects of this treatment reported on day 12. For PC-GqD–KO mice, telemeters were implanted on day –3, with mice maintained with standard chow until day 0. Starting blood pressures were reported on day 0. On day 1, mice were switched to the DOCA-salt regimen for the next 20 days. The effects of DOCA-salt were reported day 10. Daily CNO injections began day 15 with effects reported on day 20.

### Statistics.

Data were analyzed and plotted using GraphPad Prism 9 (GraphPad Software, Inc.). Summarized data reported as mean ± SEM. Summarized data were compared with the Student’s (2-tailed) *t* test or a 2-way ANOVA. *P* ≤ 0.05 was considered significant.

### Study approval.

The University of Texas Health Science Center at San Antonio approved all animal procedures. All animal use and welfare adhered to the NIH *Guide for the Care and Use of Laboratory Animals* (National Academies Press, 2011) following protocols reviewed and approved by the Institutional Animal Care and Use Committee of the University of Texas Health Science Center at San Antonio.

## Author contributions

AGS, TMAEA, and JDS designed the research studies. AGS, TMAEA, JC, and EM conducted experiments and acquired data. AGS, TMAEA, and JDS analyzed data. AGS, TMAEA, CRA, and JDS wrote the manuscript.

## Version 1 06/06/2023

In-Press Preview

## Version 2 07/24/2023

Electronic publication
